# Intratumoural renal cell carcinoma haemorrhage following stereotactic radiotherapy: a case report

**DOI:** 10.1186/s12885-019-5899-3

**Published:** 2019-07-08

**Authors:** Liam A. Hilleary, Christopher Wratten, Shankar Siva, Jenna Hilleary, Jarad M. Martin

**Affiliations:** 1 Calvary Mater Hospital, Locked Mail Bag 7 Hunter Region Mail Centre, Newcastle, New South Wales 2310 Australia; 2 University of Newcastle School of Medicine and Public Health, University Drive, Callaghan, New South Wales 2308 Australia; 3 Peter MacCallum Cancer Centre, Locked Bag 5m A’Beckett St, Melbourne, Victoria 8006 Australia

**Keywords:** Renal cell carcinoma, Stereotactic body radiotherapy, Spontaneous bleeding, Intratumoural bleeding

## Abstract

**Background:**

Stereotactic radiotherapy is an emerging treatment option for patients with inoperable renal cell carcinoma (RCC). Haemorrhage has not previously been reported to occur as a result of Stereotactic Body Radiotherapy (SBRT) to the kidney for primary RCC. We report an acute haemorrhage in a patient who received only one of three planned fractions of SBRT as part of a clinical trial.

**Case presentation:**

A 74 year old female had a left renal mass under observation for 4 years, during which time she was imaged repeatedly using ultrasound and CT scans. There has been no evidence of metastases, and the lesion has demonstrated a steady pattern of growth over the 4-year period. Fine needle aspiration histologically confirmed RCC.

Following a multidisciplinary review, the patient was recommended for SBRT as she was not considered a surgical candidate. Treatment was planned for an ablative 42Gray (Gy) to be delivered in 3 fractions at 14Gy/fraction as part of a clinical trial. Our patient presented to the emergency department (ED) suffering left flank pain, fever and vomiting within 3 h of the first fraction of SBRT. CT showed the mass to have markedly increased in size, measuring 8.7 × 8.1 × 7.0 cm, from 6.5 × 5.4 × 5.6 cm. It was reported as an internal haemorrhage into the malignancy. The patient was admitted for analgesia, anti-pyretics, and transfusion of 2 units of packed red blood cells. The patient recovered without any further intervention but radiotherapy was discontinued. The patient was alive and free from disease progression two years after the aborted treatment.

**Conclusion:**

Such events, though rare, are potentially serious, and therefore clinicians should be aware of such treatment related complications.

## Background

Either partial or radical nephrectomy is the standard treatment for primary renal cell carcinoma (RCC) [1]. Thermal ablation approaches like radiofrequency ablation (RFA) or Cryotherapy are alternatives for small renal masses. Stereotactic Body Radiotherapy (SBRT) uses highly focused beam of X-Rays to cause genetic damage to cells, and has become a standard approach for treating a range of cancers including early primary lung tumours and brain metastases [2, 3]. More recently, SBRT has been investigated as another approach of ablating inoperable abdominal tumours, including RCC, and has the advantage of being able to treat larger renal tumours [4, 5] due to its generous beam geometry when compared to the smaller ablation zone inflicted by microwave and radiofrequency ablation. Indeed, a pooled analysis of 223 patients managed with SBRT showed a 2 year rate of freedom from local progression of 100%, justifying ongoing research in this area [4].

Due to their hypervascular nature, spontaneous haemorrhage has been reported in cases of RCC. Spontaneous haemorrhage can present as a symptom in the presentation of RCC, often characterised by flank pain and fever. In the treatment setting, bleeding is more commonly associated with invasive approaches such as radiofrequency ablation where a large study demonstrated haemorrhage in almost 6% of patients [6], or systemically administered targeted therapies like sorafenib, where it has been linked to fatal haemorrhage in the treatment of central lung tumours [7]. Here we present an unusual but serious adverse haemorrhage in a patient receiving SBRT to a primary RCC of the kidney.

## Case presentation

A 74 year old female had a left renal mass under observation for 4 years, during which time she was imaged repeatedly using ultrasound and CT scans. Previous medical history includes obesity, diverticulosis, paroxysmal atrial fibrillation, diabetes mellitus, hypertension, and chronic kidney disease. Relevant medications included aspirin 100 mg/day, which was not ceased. She ceased smoking 30 years ago. In December 2012, the lesion measured 39 mm in its largest dimension, and in July 2016 was measured at 65 mm.There has been no evidence of metastases, and the lesion has demonstrated a steady pattern of growth over the 4-year period. Fine needle aspiration performed 3 months prior to presentation histologically confirmed the mass to be consistent with RCC.

Following a multidisciplinary review, the patient was recommended for SBRT as she was not considered a surgical candidate. The tumour was not amenable to thermal ablation due to size. She signed Informed Consent for a Human Research Ethics Committee approved clinical trial [5]. Treatment was planned for 42Gy to be delivered in 3 fractions at 14Gy per fraction as part of a prospective phase 2 collaborative clinical trial [5]. Non-contrast 3D and 4D CT scans were performed with 2 mm slice thickness. A recent contrast enhanced diagnostic CT was fused to the 3D planning CT.

**Fig. 1:**
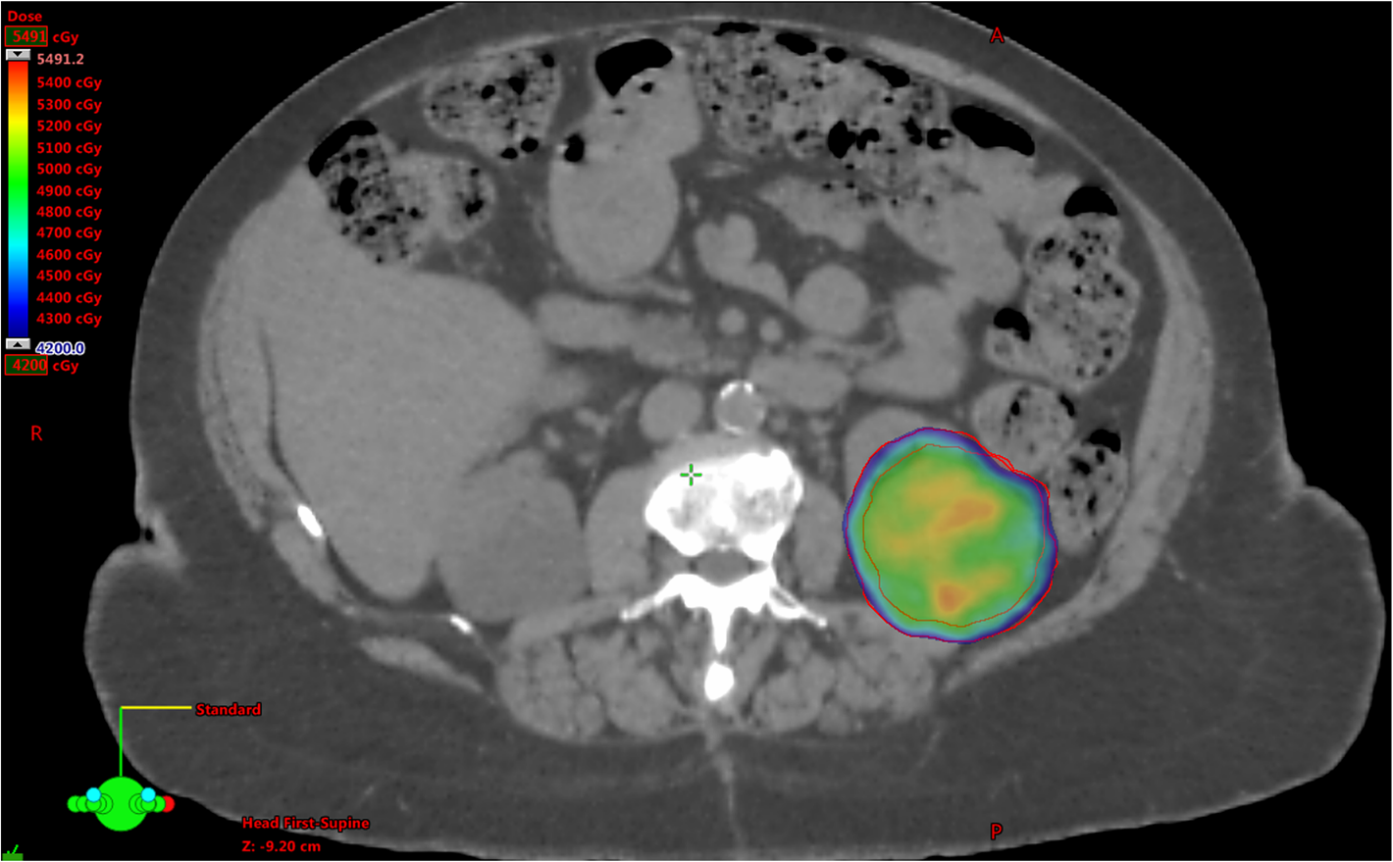
legend: Axial view of dosimetry as seen on planning CT demonstrates doses ranging from 42Gy. Doses greater than 42Gy are restricted to the planning target volume (outer contour), and the dose was reduced in areas that overlap bowel. Note that due to the haemorrhage, treatment was suspended after 14Gy had been delivered

A gross tumour volume (GTV) was contoured on the co-registered 3D CT scan, measuring 65 mm in its largest dimension. An internal target volume (ITV) was created by using 4D CT to account for breathing motion (Fig. 1). Motion observed was minimal, reaching 5 mm cranio-caudally, and 3 mm anterio-laterally. There was no posterior or medial motion observed. The planning target volume (PTV) was a 6 mm expansion of the ITV, optimised to remove regions overlapping with large bowel, with the ITV being used to define PTV extent in the region of overlap.

Varian Eclipse planning system was used for dose calculation and treatment was delivered using 10MV Flattening Filter Free (FFF) VMAT consisting of two coplanar, half rotation arcs, avoiding the right abdominal region.

A cone beam computed tomography scan (CBCT) was performed pre, mid, and post treatment, with corrections for intra-fractional motion being made prior to the delivery of each arc. Image matching was performed by two Radiation Therapists in consultation with the Radiation Oncologist. The GTV was easily defined on CBCT, as were proximal structures such as bowel and the renal artery and vein.

**Fig. 2:**
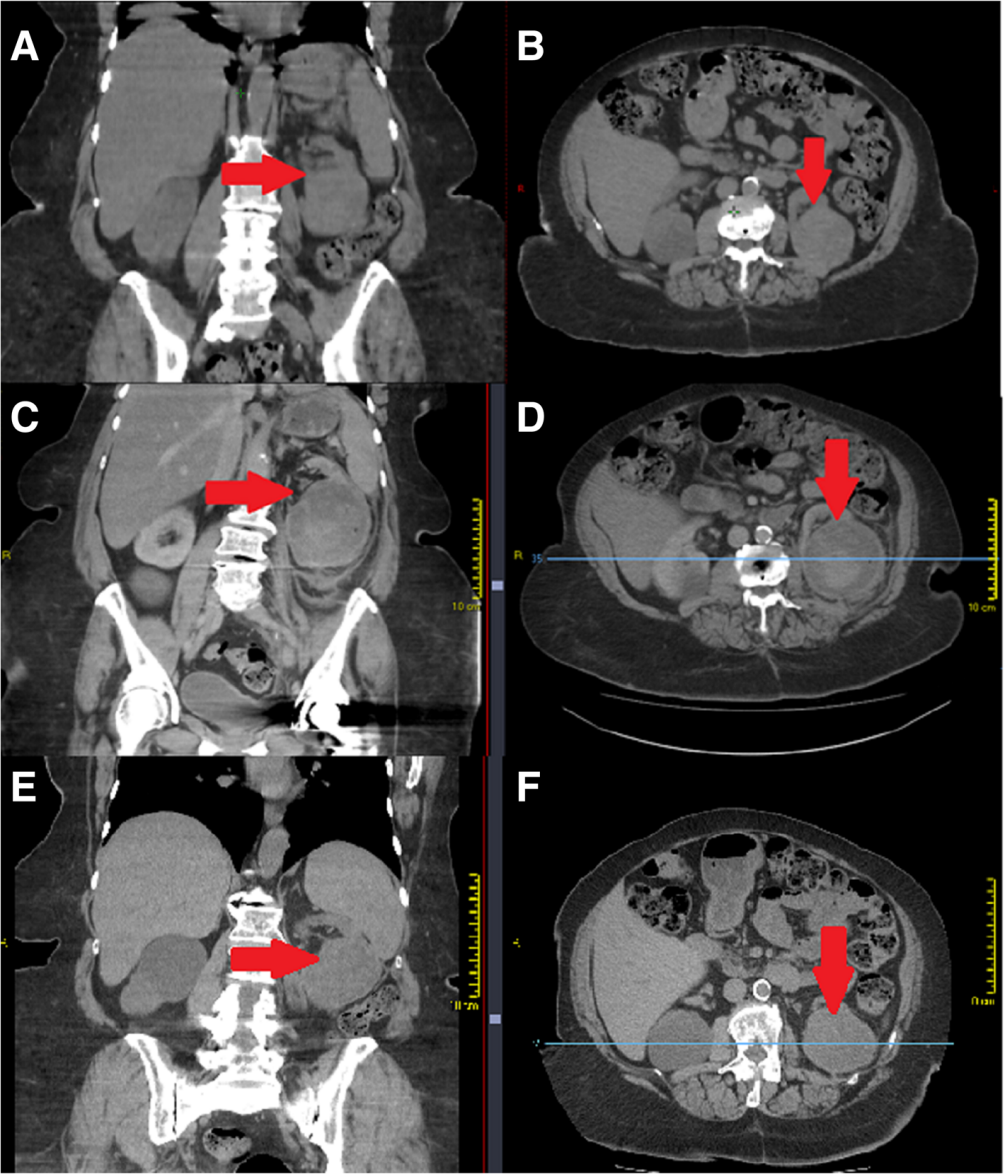
legend: RCC mass measuring 65 mm in its largest dimension at the time of planning CT (images **a**,**b**), compared to 87 mm mass with internal haemorrhage as seen in diagnostic CT after first of three prescribed ablative radiotherapy fractions (images **c**, **d**), and 60 mm at 18 months following adverse event (images **e**, **f**)

Within 3 h following the first fraction of SBRT, this patient presented to the emergency department suffering severe left flank pain, fever and vomiting. Contrast enhanced CT showed the mass to have markedly increased in size, measuring 87 × 81 × 70 mm, compared with 65 × 54 × 56 mm earlier the same day on CBCT which had been stable compared with her planning CT (Fig. 2). Intratumoral haemorrhage was noted with extension into the intraperitoneal space. The patient was admitted for analgesia, anti-pyretics, and transfusion of 2 units of packed red blood cells. The patient recovered without need for any further intervention but given this adverse reaction, radiotherapy was discontinued after 14Gy as a single fraction. The patient remained in hospital for observation for a total of 8 days, and screening for coagulopathy was negative. Two years after the event the patient is stable, CT imaging showing a reduced mass measuring 55 mm in maximal diameter, similar to pre-treament imaging measurements on the day of radiotherapy, and suggesting on-going fibrosis and disease response. No metastatic lesions are evident, and dimercapto succinic acid (DSMA) split renal function scan shows 35% of function in the left kidney and an increasing estimated glomular filtration rate (eGFR) now to a level of 40.

## Discussion

We report a patient with primary RCC who experienced an intratumoral haemorrhage immediately after receiving part of a planned ablative course of radiation therapy. Both in the preceding single centre study, and a larger multi-centre report of 223 patients, no similar events had been observed [4, 5]. Research in animal cancer models has suggested that endothelial cells are relatively resistant to radiation induced apoptosis [6]. Never the less, haemorrhage has been reported as a possible complication of cranial and pulmonary stereotactic radiotherapy (SRT) due to radionecrosis [7–9]. However, in the general oncology setting haemorrhage is often controlled, not caused, by radiotherapy. Hence radiotherapy is widely accepted as a standard treatment for many sites of haemorrhage associated with advanced cancer [10, 11].

Radionecrosis may be influenced by increased vasculature as well as increased radiosensitivity bought on by additional systemic therapies [7–11], especially when combined with hypofractionated radiotherapy. Our patient did not partake in such systemic therapies, however conditions such as diabetes, chronic renal insufficiency, and hypertension have been linked to reduced tolerance to radiotherapy [12, 13], and the pathways that lead to tumour shrinkage could disrupt the microvascular structure in a way that contributes to spontaneous or gradual bleeding. A magnetic resonance imaging study on the incidence of haemorrhage in patients with chronic renal insufficiency demonstrated that 12 of 13 consecutive patients exhibited intratumoral haemorrhage [14], an illustration of the fragile stroma of primary renal cell tumours. The precise mechanism of endothelial cell function in the face of radiation is very complex, including an array of cytokines, inherent endothelial cellular changes and interaction with circulating immune cells.

Thrombotic lesions can be produced following the release of cytokines when injury to the endothelial cells has occurred, and this has been used to explain bleeding events after cranial radiosurgery where haemorrhage is more frequently reported in the literature. Other hypotheses related to acute and late cranial bleeds include high feeding artery pressures and the increase in intravascular outflow resistance caused by venous obliteration, and more simply, there is the likelihood that large, vessel-rich tumours that were more likely to bleed if left untreated, will remain more likely to bleed even when treated [15–17]. Some possible causes stated in the literature are less relevant to body radiotherapy such as disruption to the blood brain barrier and the presence of pre-existing aneurisms proximal to the target region [15, 16]. Most commonly, post-treatment bleeding occurs as a delayed effect in the treatment of arteriovenous malformations (AVM), with a latency period of between 6 months and 3 years [16].In the intracranial metastasis setting, it should be noted that subacute intracranial bleeds following radiotherapy are mostly reported to have occurred over a longer time interval, with a study showing 10 intracranial tumour bleeds among 10 patients with metastatic lesions to have an interval period ranging from 1 day to 4 months [15].

We hypothesize that acute effects of the high dose of radiation on abnormal tumour endothelial cells in conjunction with mild anticoagulation in the form of aspirin was the mechanism behind the observed haemorrhage.

Although our patient was stabilised following haemorrhage, the required acute procedures such as blood transfusion carry additional risks such as infection and poorer response to medical treatments [18, 19]. Validated tools such as the HAS-BLED (Hypertension, Abnormal Renal/Liver Function, Stroke, Bleeding History or Predisposition, Labile INR, Elderly, Drugs/Alcohol Concomitantly) scoring criteria may be considered to assist with risk stratification [20–23]. Participant informed consent for the current study has been altered to reflect haemorrhage as a rare potential toxicity.

## Conclusion

Given the timing relative to treatment delivery, we believe the acute haemorrhage to be a radiation-related effect. We hypothesize that endothelial disruption led to acute oedema and subsequent rupture of friable tumour neovasculature [24]. No similar events have been reported in the literature for primary extracranial lesions. Although unlikely, it is recommended that this risk is disclosed as part of informed consent, and that patients are aware of the experimental nature of SBRT for this indication.

## Data Availability

Not applicable.

## Abbreviations

3D: Three dimensional
4D: Four dimensional
AVM: Arteriovenous malformation
CBCT: Cone beam CT
CT: Computed tomography
DSMA: Dimercapto succinic acid
eGFR: Estimated glomular filtration rate
FFF: Flattening filter free
GTV: Gross tumour volume
Gy: Gray
ITV: Internal target volume
MV: Megavolt
PTV: Planning target volume
RCC: Renal cell carcinoma
RFA: Radiofrequency ablation
SBRT: Stereotactic body radiotherapy
